# The Risk Factor Analysis of Gallbladder Gangrene in Acute Acalculous Cholecystitis: A Single-Center Retrospective Study

**DOI:** 10.1155/grp/8696872

**Published:** 2025-10-21

**Authors:** Jiu-ling Zheng, Shuang-quan Liu, Yan-han Liu, Guo-hua Dai, Hua-guo Feng, Hao-yang Tan

**Affiliations:** ^1^Department of Hepatobiliary Surgery, The Chongqing University Jiangjin Hospital, School of Medicine, Chongqing University, Chongqing, China; ^2^Department of Radiology, The Chongqing University Jiangjin Hospital, School of Medicine, Chongqing University, Chongqing, China

**Keywords:** acute acalculous cholecystitis, gallbladder gangrene, inflammatory markers, risk factors, systemic immune-inflammation index

## Abstract

**Objective:**

This research was performed to determine the risk factors for gallbladder gangrene in acute acalculous cholecystitis patients and to assess the predictive ability of inflammatory markers.

**Methods:**

The study included 226 acute acalculous cholecystitis patients who underwent laparoscopic cholecystectomy within 72 h of onset. The receiver operating characteristic curves were employed to determine the optimal cut-off, specificity, and sensitivity of inflammatory markers in predicting gangrenous cholecystitis. Logistic regression analysis was conducted to ascertain the independent risk factors associated with gallbladder gangrene.

**Results:**

The incidence rate of gallbladder gangrene in acute acalculous cholecystitis was 45.1% (102/226). Compared with other inflammatory markers, the systemic immune-inflammation index (SII) demonstrated superior predictive validity (vs. CRP, *p* = 0.021; vs. WBC, *p* < 0.001; vs. PCT, *p* = 0.004; vs. NLR, *p* < 0.001; vs. PLR, *p* < 0.001). The results of the logistic regression analysis revealed that platelet, PCT, SII, D-dimer, CA19-9, gallbladder enlargement, and gallbladder effusion were independent risk factors.

**Conclusion:**

This study found that platelet, PCT, SII, D-dimer, CA19-9, gallbladder enlargement, and gallbladder effusion were independent risk factors for gallbladder gangrene in acute acalculous cholecystitis. SII could serve as a novel, straightforward, and potent predictive indicator for gallbladder gangrene in acute acalculous cholecystitis.

## 1. Introduction

Acute cholecystitis is a common biliary system disease. Gangrenous cholecystitis is a severe type of acute cholecystitis, which is characterized by obvious infection and necrosis of the gallbladder wall [1]. If gangrenous cholecystitis is not treated in time, it may cause a series of serious complications, such as gallbladder perforation, biliary peritonitis, and abscess around the gallbladder [2]. These complications not only increase the difficulty of treatment but also may threaten the lives of patients [3]. However, the timing of surgery for acute acalculous cholecystitis is controversial. If there is a high risk for developing gangrene, emergency surgery may be the best treatment option. Therefore, early identification and accurate evaluation of gangrenous cholecystitis have important clinical significance for optimizing the treatment plan and improving the prognosis in acute acalculous cholecystitis.

Based on the recommendation outlined in the Tokyo Guidelines 2018 [4], the white blood cell (WBC) count can serve as an indicative marker for assessing the severity of acute cholecystitis. Some research showed that the WBC count of patients with gangrenous cholecystitis is higher than that of patients with acute cholecystitis, and 95% of gangrenous cholecystitis' WBC count > 10∗10^9^/L [5, 6]. However, due to the lack of a unified diagnostic standard for WBC count, it is difficult to promote its application in clinical practice. C-reactive protein (CRP), platelet-to-lymphocyte ratio (PLR), and neutrophil-to-lymphocyte ratio (NLR) are additionally recognized as common inflammatory markers in clinical practice. A retrospective study showed that the increase in CRP suggested that the histological state of the gallbladder was poor, which was helpful in identifying gangrenous cholecystitis [7]. When CRP rose to 200 mg/L, the positive predictive value for predicting gangrenous cholecystitis was 50%, and the sensitivity and negative predictive value were 100%. CRP rise was also an effective inflammatory marker for emergency surgery and laparoscopic cholecystectomy converted to open surgery [8]. Recent research showed that NLR in patients with gangrenous cholecystitis will also be significantly increased. CRP and NLR are independent related factors of the severity of acute cholecystitis, which can help the diagnosis and prognosis evaluation of gangrenous cholecystitis [9]. Some studies found a correlation between the inflammatory indices, such as PLR, and the severity of inflammation and postoperative hospital stay in acute calculous cholecystitis [10, 11]. However, the relationship between these inflammatory indices and predicting gangrenous cholecystitis in acute acalculous cholecystitis lacks relevant research.

More and more researchers believe that the hypercoagulable state of elevated platelets can also reflect the severity of the systemic inflammatory response. In recent years, some scholars have proposed a new parameter, which is named “systemic immune-inflammation index (SII)” [12]. SII is a new indicator for integrating neutrophils, lymphocytes, and platelet cell counts, which can better assess the severity of inflammation and blood hypercoagulability. Recent studies have shown that SII can help to distinguish the risk of acute cholecystitis and chronic cholecystitis and can predict the severity of acute cholecystitis [13]. The relationship between SII and acute acalculous gangrenous cholecystitis deserves further investigation.

Therefore, the aim of this study was to determine the risk factors for gallbladder gangrene in acute acalculous cholecystitis patients and to assess the predictive ability of inflammatory markers.

## 2. Materials and Methods

### 2.1. Patients

This retrospective study included acute acalculous cholecystitis patients who underwent laparoscopic cholecystectomy at the hepatobiliary surgery department from January 2021 to August 2024. The inclusion criteria were as follows: (1) age exceeding 18 years old; (2) the diagnostic criteria of acute acalculous cholecystitis were according to the Tokyo Guidelines 2018; (3) patients underwent laparoscopic cholecystectomy within 72 h of onset and had a detailed gallbladder pathology report after surgery; (4) patients signed informed consent before receiving treatment; and (5) clinical data were comprehensively recorded. The exclusion criteria were as follows: (1) patients with gallstones, choledocholithiasis, or pancreatitis; (2) patients with malignant tumors such as liver cancer or gallbladder cancer; and (3) patients with serious underlying diseases such as organ failure. Eligible patients were divided into the gangrene group and the non-gangrene group based on gallbladder pathological results.

### 2.2. Data Collection

From electronic medical record systems, we have compiled the following clinical data: age, gender, vital signs, underlying disease, blood routine tests, coagulation function data, liver and kidney function data, inflammatory cytokine data, imaging examination, and treatment process. Abdominal ultrasound and/or abdominal CT, judged by two senior ultrasound physicians or radiologists, were used to document the thickness of the gallbladder walls, enlarged gallbladder, and pericholecystic fluid collection. The most severe values of laboratory and imaging examination data recorded within 24 h before surgery were selected.

Inflammatory biomarkers were computed utilizing the blood routine test results measured from the same sample (such as neutrophil count, lymphocyte count, and platelets). PLR was derived using the following formula: PLR = platelets/lymphocyte count. Similarly, NLR was computed with the following formula: NLR = neutrophil count/lymphocyte count. SII was calculated employing the following formula: SII = platelets∗neutrophil count/lymphocyte count.

The sample size of this study was calculated by the calculation method of Li and Fine [14].

### 2.3. Statistical Analysis

The SPSS Statistics software (Version 23, New York, United States) was used for statistical analysis in this retrospective study. Continuous data were presented as medians accompanied by interquartile ranges (25th–75th percentile) or as means ± standard deviations. These data were subsequently subjected to comparative analysis utilizing either the Mann–Whitney *U* test or the Student's *t*-test according to whether they conform to a normal distribution. Dichotomous data were presented as frequencies (percentages) and compared by the chi-square or Fisher's exact test as appropriate. The receiver operating characteristic (ROC) curves were employed to determine the optimal cut-off, specificity, and sensitivity of inflammatory biomarkers in predicting gangrenous cholecystitis. The area under the curve (AUC) was used to verify predictive performance and was categorized according to the widely accepted classification scale described by Micić et al. [15]. The differences between inflammatory indicators' AUC were analyzed by the calculation method of DeLong et al. [16]. Univariate analysis for each factor was performed by logistic regression analysis. The continuous variable was dichotomized based on optimal cut-off values by ROC curve. Variables with *p* values less than 0.1 in univariate analysis were included in the multivariate regression model using the “forward LR” method to determine independent risk factors. The two-tailed *p* value < 0.05 was considered a statistically significant difference.

## 3. Results

### 3.1. Baseline Characteristics

**Figure 1:**
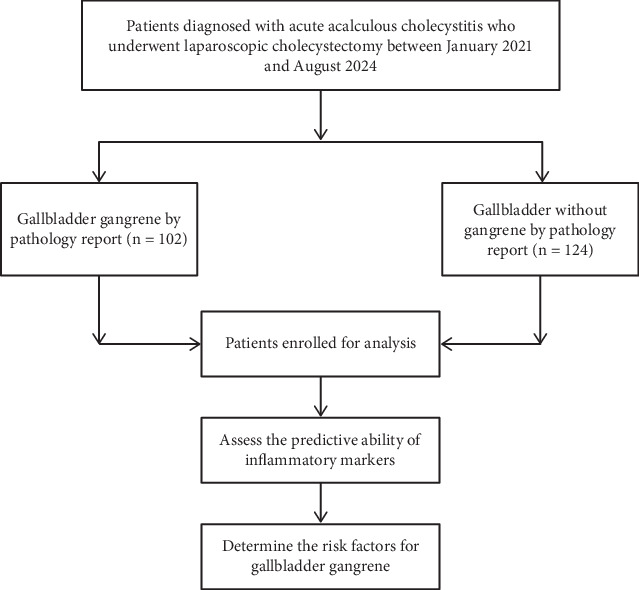
Research flowchart.

There were 226 patients with acute acalculous cholecystitis who underwent laparoscopic cholecystectomy within 72 h of onset included in this study. According to postoperative pathological reports, these patients were categorized into two groups: the gangrene group (*n* = 102) and the non-gangrene group (*n* = 124), as shown in Figure 1.

The incidence rate of gangrenous cholecystitis in acute acalculous cholecystitis was 45.1% (102/226). Compared with non-gangrene group patients, the gangrene group patients' median age was older (61.0 vs. 55.5, *p* = 0.001). The inflammatory markers (CRP, WBC, PCT, NLR, PLR, and SII) of the gangrene group were higher than those of the non-gangrene group. The levels of D-dimer and carbohydrate antigen 19-9 (CA19-9) between the two groups were statistically different. There were also statistical differences in gallbladder ultrasound between the two groups. The median gallbladder wall of gangrene group patients was thicker than that of non-gangrene group patients (0.5 vs. 0.3 cm, *p* < 0.001). The incidence rates of gallbladder enlargement and effusion in the gangrene group were higher than those in the non-gangrene group (63.7% vs. 18.5%, *p* < 0.001; 31.4% vs. 1.6%, *p* < 0.001).

There were also notable statistical differences observed in the perioperative conditions between the two groups. The median operative time and postoperative hospitalization time of gangrene group patients were longer than those of non-gangrene group patients (71.5 vs. 50.5 min, *p* < 0.001; 3 vs. 2 days, *p* < 0.001). The median intraoperative bleeding of gangrene group patients was more than that of non-gangrene group patients (30.0 vs. 10.0 mL, *p* < 0.001). The conversion rate of gangrene group patients was higher than that of non-gangrene group patients (5.9% vs. 0.0%, *p* = 0.020).

The baseline characteristics of the two groups of patients are outlined in Table 1.

### 3.2. Prediction Value of Inflammatory Markers

**Figure 2:**
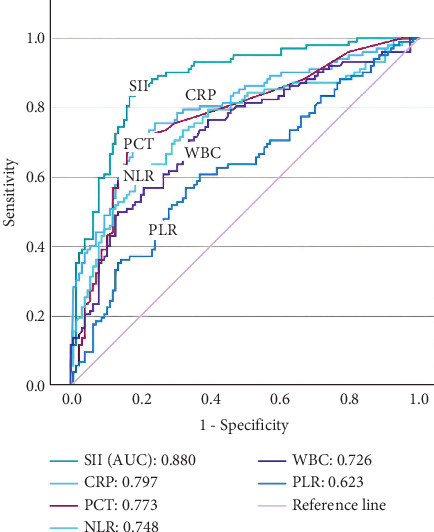
The receiver operating characteristic curves of the inflammatory biomarkers for gallbladder gangrene in acute cholecystitis patients. SII, systemic immune-inflammation index; CRP, C-reactive protein; WBC, white blood cell count; PCT, procalcitonin; NLR, neutrophil-to-lymphocyte ratio; PLR, platelet-to-lymphocyte ratio; AUC, area under the curve.

Furthermore, the predictive values of the inflammatory markers (CRP, WBC, PCT, NLR, PLR, and SII) for gallbladder gangrene in acute acalculous cholecystitis patients were comprehensively analyzed utilizing the ROC curves (Figure 2). The AUC for SII was determined to be 0.880 (*p* < 0.001), and the cut-off threshold was established at 1659.0∗10^9^/L. The AUC for CRP was determined to be 0.797 (*p* < 0.001), and the cut-off threshold was established at 19.02 mg/L. The AUC for WBC was determined to be 0.726 (*p* < 0.001), and the cut-off threshold was established at 7.72∗10^9^/L. The AUC for PCT was determined to be 0.773 (*p* < 0.001), and the cut-off threshold was established at 0.12 ng/mL. The AUC for NLR was determined to be 0.748 (*p* < 0.001), and the cut-off threshold was established at 7.28. The AUC for PLR was determined to be 0.623 (*p* = 0.001), and the cut-off threshold was established at 199.11. Compared with other inflammatory markers, SII had a larger AUC that demonstrated superior predictive validity (vs. CRP, *p* = 0.021; vs. WBC, *p* < 0.001; vs. PCT, *p* = 0.004; vs. NLR, *p* < 0.001; vs. PLR, *p* < 0.001; Table 2).

Based on the sensitivity and specificity of SII (*α* = 0.05, allowance error was less than 0.05), the sample size of this study was calculated as 170 patients.

### 3.3. Risk Factor Analysis

The results of the univariate and multivariate logistic regression analysis revealed that platelet, PCT, SII, D-dimer, CA19-9, gallbladder enlargement, and gallbladder effusion were independent risk factors of gallbladder gangrene in acute acalculous cholecystitis patients. The comprehensive findings are presented in Table 3.

## 4. Discussion

Gangrenous cholecystitis is a common severe acute cholecystitis in clinical practice. Its condition progresses rapidly and has a high mortality rate. Once diagnosed as missed, it may cause serious consequences. Our study found that the operative time, intraoperative blood loss, conversion to open surgery rate, and postoperative hospitalization time of gangrenous cholecystitis patients were higher than those of non-gangrene cholecystitis patients. It is particularly important to early identify acute acalculous cholecystitis patients at high risk of gallbladder gangrene and take timely treatment. Recently, some studies have found the factors for predicting gallbladder gangrene, such as age, gender, inflammatory markers, and gallbladder ultrasound [17–19]. CA19-9, as a gastrointestinal cancer-related antigen, is not only highly expressed in pancreatic cancer and biliary tract cancer but also occasionally detected in benign biliary diseases, such as cholelithiasis, cholecystitis, and cholangitis [20, 21]. Our study revealed that the levels of CA19-9 in gangrene cholecystitis patients were higher than those in non-gangrene cholecystitis patients. The multivariate analysis result also confirmed that the high level of CA19-9 was an independent risk factor for gallbladder gangrene in acute acalculous cholecystitis.

The gallbladder imaging examination also plays an important role in the diagnosis of gangrenous cholecystitis. A recent study reported that gallbladder wall edema, gallbladder dilation, and fluid accumulation around the gallbladder should be used as predictive indicators for gangrenous cholecystitis [22]. Our study found that compared with non-gangrenous cholecystitis patients, gangrenous cholecystitis patients had thicker gallbladder walls. Gallbladder enlargement and gallbladder effusion were independent risk factors for gallbladder gangrene. Local special changes in the gallbladder often indicate the presence of gallbladder gangrene. Some studies showed that contrast-enhanced ultrasound, computerized tomography scan, and magnetic resonance imaging can also be used as supplementary inspection methods for gangrenous cholecystitis [8, 19, 23, 24].

This retrospective study indicated that the inflammatory markers were important factors in predicting gallbladder gangrene in acute acalculous cholecystitis patients. Compared with non-gangrenous cholecystitis patients, the inflammatory markers of gangrenous cholecystitis patients were significantly increased. These inflammatory markers had demonstrated good predictive ability for gallbladder gangrene. Compared with other inflammatory markers, SII had a higher predictive ability. The SII performed best for predicting gallbladder gangrene in acute acalculous cholecystitis patients. The multivariate analysis results showed that PCT and SII were the independent risk factors of gallbladder gangrene. Other inflammatory indicators were not independent risk factors of gallbladder gangrene, though the inflammatory indicators were increased in gangrenous cholecystitis. Compared to other inflammatory markers, SII comprehensively reflects the inflammatory state and blood hypercoagulability. Our study revealed that high levels of platelet and D-dimer were the independent risk factors of gallbladder gangrene. It seems to suggest that local blood circulation disorder and microthrombus formation may be the important factors leading to gallbladder gangrene in acute acalculous cholecystitis. For acute acalculous cholecystitis patients, we should pay more attention to changes in local circulatory function rather than inflammatory response. We speculate that early anticoagulant therapy and improving microcirculation (within 72 h of onset) may reduce the risk of developing gallbladder gangrene. It requires further research to confirm this hypothesis and explore potential mechanisms.

Some limitations need to be pointed out in the current research. First, the retrospective and single-center nature of our study limits causal inference and generalizability. Selection bias is inherent as we only included patients who underwent surgery within a specific timeframe, excluding those managed conservatively or with drainage, potentially underestimating the true incidence and spectrum of gallbladder gangrene. Second, the diagnosis of gangrene relied solely on postoperative pathology, leaving the status of nonsurgical patients unknown and introducing diagnostic verification bias. Third, due to retrospective data constraints, we were unable to include important inflammatory cytokines such as IL-6 and TNF-*α*, which might offer additional predictive value. Fourth, although we performed a sample size calculation, our cohort remains relatively modest, potentially limiting the power to detect smaller effect sizes or less common risk factors. The dichotomization of continuous variables, while practical for clinical application, may oversimplify complex biological relationships. Fifth, despite multivariate adjustment, residual confounding from unmeasured factors such as detailed sepsis criteria, prior treatments (antibiotics, anticoagulants), and granular comorbidity severity could influence our results. Finally, our study focused on short-term perioperative outcomes; long-term follow-up data regarding recurrence, survival, or quality of life are lacking. Future prospective, multicenter studies incorporating a broader range of biomarkers, including cytokines, and including both surgical and nonsurgical patients with long-term follow-up are warranted to validate and extend our findings.

## 5. Conclusion

This study found that platelet, PCT, SII, D-dimer, CA19-9, gallbladder enlargement, and gallbladder effusion were independent risk factors for gallbladder gangrene in acute acalculous cholecystitis. Compared with other inflammatory markers, SII demonstrated a superior predictive efficacy. SII could serve as a novel, straightforward, and potent predictive indicator for gallbladder gangrene in acute acalculous cholecystitis.

## Figures and Tables

**Table 1:** Baseline characteristics of acute acalculous cholecystitis patients.

	**Non-gangrene (** **n** = 124**)**	**Gangrene (** **n** = 102**)**	**p** **value**
Gender (%)			0.179
Male	45 (36.3)	46 (45.1)	
Female	79 (63.7)	56 (54.9)	
Age, median (Q1, Q3)	55.5 (47.7, 65.3)	61.0 (53.0, 72.0)	0.001⁣^∗^
BMI, mean ± SD	24.8 ± 3.8	25.3 ± 3.8	0.421
Underlying disease (%)			0.445
None	86 (69.4)	70 (68.6)	
Hypertension	21 (16.9)	16 (15.7)	
Diabetes	11 (8.9)	6 (5.9)	
Other	6 (4.8)	10 (9.8)	
CRP (mg/L), median (Q1, Q3)	5.7 (52.2, 18.1)	117.5 (12.4, 200.0)	< 0.001⁣^∗^
PLT (∗10^9^/L), mean ± SD	222.57 (62.53)	218.22 (70.30)	0.623
WBC (∗10^9^/L), median (Q1, Q3)	6.8 (4.6, 9.7)	10.5 (7.8, 13.1)	< 0.001⁣^∗^
PCT (ng/mL), median (Q1, Q3)	0.05 (0.03, 0.09)	0.27 (0.08, 1.17)	< 0.001⁣^∗^
NLR, median (Q1, Q3)	5.4 (3.2, 8.8)	10.3 (6.8, 15.6)	< 0.001⁣^∗^
PLR, median (Q1, Q3)	168.3 (125.3, 234.3)	215.9 (138.1, 290.1)	0.002⁣^∗^
SII (∗10^9^/L), median (Q1, Q3)	989.2 (605.3, 1499.7)	2357.8 (1833.3, 3071.5)	< 0.001⁣^∗^
TB (*μ*mol/L), median (Q1, Q3)	12.1 (8.5, 16.6)	14.6 (8.5, 21.4)	0.105
ALB (g/L), median (Q1, Q3)	44.0 (41.4, 46.9)	42.9 (39.9, 45.4)	0.102
ALT (U/L), median (Q1, Q3)	21.0 (15.0, 40.5)	22.0 (16.0, 38.8)	0.841
AST (U/L), median (Q1, Q3)	21.0 (18.0, 32.5)	24.0 (19.0, 34.8)	0.517
ALP (U/L), median (Q1, Q3)	82.5 (70.0, 102.0)	89.5 (75.3, 117.5)	0.055
GGT (U/L), median (Q1, Q3)	37.0 (22.0, 79.0)	43.0 (29.3, 73.3)	0.345
CREA (*μ*mol/L), median (Q1, Q3)	664.0 (53.0, 73.3)	65.5 (55.0, 82.8)	0.185
D-dimer (*μ*g/mL), median (Q1, Q3)	0.88 (0.69, 1.15)	1.68 (1.15, 3.08)	< 0.001⁣^∗^
CA19-9 (U/mL), median (Q1, Q3)	11.9 (5.5, 18.1)	39.3 (11.5, 192.9)	< 0.001⁣^∗^
Gallbladder wall (cm), median (Q1, Q3)	0.3 (0.2, 0.5)	0.5 (0.4, 0.7)	< 0.001⁣^∗^
Gallbladder enlargement (%)	23 (18.5)	65 (63.7)	< 0.001⁣^∗^
Gallbladder effusion (%)	2 (1.6)	32 (31.4)	< 0.001⁣^∗^
Operation information			
Operative time (min), median (Q1, Q3)	50.5 (35.0, 70.0)	71.5 (59.3, 111.3)	< 0.001⁣^∗^
Intraoperative bleeding (mL), median (Q1, Q3)	10.0 (10.0, 10.0)	30.0 (10.0, 50.0)	< 0.001⁣^∗^
Conversion rate (%)	0 (0.0)	6 (5.9)	0.020⁣^∗^
Postoperative hospitalization (day), median (Q1, Q3)	2.0 (2.0, 3.0)	3.0 (2.0, 4.0)	< 0.001⁣^∗^
Complication (%)	0 (0.0)	3 (2.9)	0.181

Abbreviations: ALB, albumin; ALP, alkaline phosphatase; ALT, alanine aminotransferase; AST, aspartate aminotransferase; BMI, body mass index; CREA, creatinine; CRP, C-reactive protein; GGT, gamma-glutamyl transpeptidase; NLR, neutrophil-to-lymphocyte ratio; PCT, procalcitonin; PLR, platelet-to-lymphocyte ratio; PLT, platelet; SII, systemic immune-inflammation index; TB, total bilirubin; WBC, white blood cell count.

⁣^∗^*p* values < 0.05 were considered statistically significant.

**Table 2:** Comparison of inflammatory markers for predicting gangrenous cholecystitis.

**Variable**	**Sensitivity**	**Specificity**	**Cut-off value**	**AUC**	**95% CI**	**p** **value**	**p** **value (compared with SII)**
SII (∗10^9^/L)	0.863	0.806	1659.00	0.880	0.830–0.919	< 0.001⁣^∗^	Reference
CRP (mg/L)	0.755	0.758	19.02	0.797	0.739–0.847	< 0.001⁣^∗^	0.021⁣^∗^
WBC (∗10^9^/L)	0.765	0.605	7.72	0.726	0.663–0.783	< 0.001⁣^∗^	< 0.001⁣^∗^
PCT (ng/mL)	0.696	0.823	0.12	0.773	0.712–0.826	< 0.001⁣^∗^	0.004⁣^∗^
NLR	0.735	0.677	7.28	0.748	0.686–0.803	< 0.001⁣^∗^	< 0.001⁣^∗^
PLR	0.569	0.669	199.11	0.623	0.556–0.686	0.001⁣^∗^	< 0.001⁣^∗^

Abbreviations: AUC, area under the curve; CI, confidence interval; CRP, C-reactive protein; NLR, neutrophil-to-lymphocyte ratio; PCT, procalcitonin; PLR, platelet-to-lymphocyte ratio; SE, standard error; SII, systemic immune-inflammation index; WBC, white blood cell count.

⁣^∗^*p* values < 0.05 were considered statistically significant.

**Table 3:** Risk factor analysis of gangrene in acute acalculous cholecystitis.

	**Univariate regression**				**Multivariate regression (forward LR)**			
	**Odds ratio (OR)**	**95% CI**		**p** **value**	**Odds ratio (OR)**	**95% CI**		**p** **value**
		**Lower**	**Upper**			**Lower**	**Upper**	
Gender	0.693	0.406	1.184	0.180				
Age (> 58)	2.409	1.408	4.121	0.001				
BMI (> 22.8)	1.764	0.952	3.268	0.071				
CRP (> 19.02)	9.651	5.242	17.767	< 0.001				
PLT (> 351)	5.191	1.077	25.020	0.040	31.704	2.126	472.794	0.012
PCT (> 0.12)	10.619	5.685	19.833	< 0.001	4.932	1.569	15.501	0.006
WBC (> 7.72)	4.974	2.779	8.904	< 0.001				
NLR (> 7.28)	5.833	3.269	10.408	< 0.001				
PLR (> 199.11)	2.669	1.552	4.587	< 0.001				
SII (> 1659.0)	26.190	12.765	53.736	< 0.001	20.764	6.537	65.959	< 0.001
TB (> 16.6)	2.284	1.293	4.033	0.004				
ALB (< 44.6)	1.510	0.881	2.587	0.134				
ALT (> 24)	1.407	0.828	2.393	0.207				
AST (> 21)	1.587	0.934	2.696	0.088				
ALP (> 74)	1.880	1.054	3.354	0.033				
GGT (> 31)	1.886	1.088	3.271	0.024				
CREA (> 74)	2.146	1.188	3.876	0.011				
D-dimer (> 1.18)	9.061	4.947	16.597	< 0.001	4.026	1.299	12.477	< 0.001
CA19-9 (> 18.8)	7.500	4.143	13.576	< 0.001	7.213	2.419	21.510	< 0.001
Gallbladder wall (> 0.4)	4.889	2.777	8.606	< 0.001				
Gallbladder enlargement	7.714	4.206	14.150	< 0.001	6.473	2.194	19.100	< 0.001
Gallbladder effusion	27.886	6.486	119.891	< 0.001	23.979	2.792	205.943	0.004

*Note:* Variables with *p* value < 0.10 were included in multivariate analysis. The statistical significance was accepted at *p* < 0.05.

Abbreviations: ALB, albumin; ALP, alkaline phosphatase; ALT, alanine aminotransferase; AST, aspartate aminotransferase; BMI, body mass index; CREA, creatinine; CRP, C-reactive protein; GGT, gamma-glutamyl transpeptidase; NLR, neutrophil-to-lymphocyte ratio; PCT, procalcitonin; PLR, platelet-to-lymphocyte ratio; PLT, platelet; SII, systemic immune-inflammation index; TB, total bilirubin; WBC, white blood cell count.

## Data Availability

The data used in the study are all included in the article and supporting information.

## Supporting Information

Supporting Information Additional supporting information can be found online in the Supporting Information section. The research process follows the STROBE guidelines. The figure legends and STROBE checklist are uploaded as Supporting Information.
